# Extended Bose-Hubbard Model with Cavity-Mediated Infinite-Range Interactions at Finite Temperatures

**DOI:** 10.1038/s41598-020-66054-1

**Published:** 2020-06-03

**Authors:** Huang-Jie Chen, Yan-Qiang Yu, Dong-Chen Zheng, Renyuan Liao

**Affiliations:** 1 Fujian Provincial Key Laboratory for Quantum Manipulation and New Energy Materials, College of Physics and Energy, Fujian Normal University, Fuzhou, 350117 China; 2 Fujian Provincial Collaborative Innovation Center for Advanced High-Field Superconducting Materials and Engineering, Fuzhou, 350117 China

**Keywords:** Ultracold gases, Physics, Atomic and molecular physics, Quantum physics

## Abstract

We consider the finite-temperature properties of the extended Bose-Hubbard model realized recently in an ETH experiment [Nature 532, 476 (2016)]. Competing short- and global-range interactions accommodate fascinating collective phenomena. We formulate a self-consistent mean-field theory to describe the behaviors of the system at finite temperatures. At a fixed chemical potential, we map out the distributions of the superfluid order parameters and number densities with respect to the temperatures. For a charge density wave, we find that the global-range interaction enhances the charge order by increasing the transition temperature at which the charge order melts out, while for a supersolid phase, we find that the disappearance of the charge order and the superfluid order occurs at different temperature. At a fixed number-density filling factor, we extract the temperature dependence of the thermodynamic functions such as internal energy, specific heat and entropy. Across the superfluid phase transition, the specific heat has a discontinuous jump.

## Introduction

The experimental progress in coupling degenerate quantum gases with light in high-Q cavities^1–9^ has opened a new avenue for creating and exploring novel many-body collective phenomena^10–12^. A paradigmatic example is the experimental realization of the Dicke model with a gas of ultracold quantum gases inside an optical cavity^4,6^, which allows for access to a superradiant phase transition associated with the breaking of a ${{\mathbb{Z}}}_{2}$ symmetry^13^. In combination with an optical lattice^14^, recent experiment has realized competing short- and long-range interactions^7^ between atoms, which accommodates a multitude of novel symmetry-broken phases, such as the charge density wave and supersolid phases. By trapping Bose-Einstein condensates inside the intersection of two high-finesse optical cavities and illuminating them by a transverse pump beam, the ETH group successfully observed supersolid formation breaking a continuous translation symmetry^8,9^.

All these exciting experimental achievements have sparked intense theoretical efforts^15–23^ concerning novel collective phenomena and dissipative dynamics arising from the cavity-mediated interactions. In particular, the extended Bose-Hubbard model realized in ETH experiment^7,24,25^ has attracted much theoretical attention. The model consists of a variation of the standard two-dimensional Bose-Hubbard model^26–29^ that includes a global-range interaction between atoms in the different checkerboard sublattices of a square lattice. It presents in total four quantum phases: superfluid (SF), supersolid (SS), Mott insulator (MI), and charge density wave (CDW). Previous theoretical studies^30–38^ mainly concentrate on the ground-state phase diagram and associated phase transitions of the model, leaving the finite-temperature physics which is experimentally relevant and interesting, largely intact.

In this work, we shall carry out a self-consistent mean-field study on the finite-temperature properties of the system, with the aim of providing qualitative predictions for future experimental investigation, as understanding even the finite-temperature properties of the conventional Bose-Hubbard model at quantitative level is a nontrivial task^39–45^. The paper is structured as follows: In Sec. II, the model is introduced and the theoretical formalism is developed. In Sec. III, we present relevant calculation results. Finally, in Sec. IV, the conclusions are drawn.

## Model and Formalism

We consider the Hamiltonian realized in the recent ETH experiment^7^ which reads

1 $$\hat{H}=-\,J\sum _{ < ij > }\,\left({\hat{b}}_{i}^{\dagger },{\hat{b}}_{j},+,h,.,c,.\right)+\frac{U}{2}\sum _{i}\,{\hat{n}}_{i}\left({\hat{n}}_{i},-,1\right)-\sum _{i}\,\mu {\hat{n}}_{i}-\frac{K}{M}{[\sum _{i}{\left(,-,,1\right)}^{{i}_{x}+{i}_{y}}{\hat{n}}_{i}]}^{2}.$$

Here *J* is the hopping amplitude between neighboring sites, *U* is the repulsive on-site contact interaction, *K* denotes the strength of global-range interaction, (*i*_*x*_, *i*_*y*_) is the coordinate of lattice site *i*, and *μ* is the chemical potential. The summation over lattice sites carries over to the total number of lattice sites *M*. The global-range interaction favors imbalanced population of bosons between even lattice sites and odd lattice sites, competing with short-range contact interaction which tends to make bosons distribute evenly among lattice sites. The interplay of three energy scales is expected to cultivate a wealth of collective phenomena.

To decouple the off-site global-range interaction, we set $\langle {(-1)}^{{i}_{x}+{i}_{y}}{\hat{n}}_{i}\rangle ={\theta }_{i}$, then by neglecting the quadratic terms in fluctuations, we obtain

2 $${[\sum _{i}{(-1)}^{{i}_{x}+{i}_{y}}{\hat{n}}_{i}]}^{2}\approx 2\sum _{i}\,{\theta }_{i}\sum _{j}\,{(-1)}^{{j}_{x}+{j}_{y}}{\hat{n}}_{j}-\sum _{i}\,{\theta }_{i}\sum _{j}\,{\theta }_{j}.$$

We proceed to introduce a charge order parameter $\Theta ={\sum }_{i}\,{\theta }_{i}/M$, which describes the average atom population difference between even and odd sites, then the term for global-range interaction becomes site-separable

$$\frac{K}{M}{[\sum _{i}{(-1)}^{{i}_{x}+{i}_{y}}{\hat{n}}_{i}]}^{2}=2K\Theta \sum _{i}\,{(-1)}^{{i}_{x}+{i}_{y}}{\hat{n}}_{i}-MK{\varTheta }^{2}.$$

To decouple the kinetic part of the Hamiltonian, we follow the usual procedures^46^ of introducing superfluid order parameters ${\psi }_{i}=\langle {\hat{b}}_{i}\rangle$, resulting in a mean-field Hamiltonian for a supercell (with one even site and one odd site):

3 $${\hat{H}}^{MF}=\sum _{s=e,o}\,\left[\frac{U}{2}{\hat{n}}_{s}\left({\hat{n}}_{s},-,1\right)-\mu {\hat{n}}_{s}\right]-2K\Theta \left({\hat{n}}_{e},-,{\hat{n}}_{o}\right)-zJ[{\psi }_{o}{\hat{b}}_{e}^{\dagger }+{\psi }_{e}^{\ast }{\hat{b}}_{o}^{\dagger }-{\psi }_{o}{\psi }_{e}^{\ast }+h.c.]+2K{\Theta }^{2},$$

where the coordination number is *z* = 2*d* with *d* being the dimension of the system, *h*.*c*. stands for a hermitian conjugate, and subindex *e* and *o* denotes even site and odd site, respectively.

We may diagonalize ${\hat{H}}^{MF}$ in the occupation number space spanned with $|{n}_{e}\rangle \otimes |{n}_{o}\rangle$ by simultaneously imposing self-consistency conditions for the charge order parameter $\Theta =\langle {\hat{n}}_{e}-{\hat{n}}_{o}\rangle /2$ and for the superfluid order parameters ${\psi }_{e}=\langle {\hat{b}}_{e}\rangle$ and ${\psi }_{o}=\langle {\hat{b}}_{o}\rangle$. It should be noted that the average of an operator $\hat{O}$ is defined as a thermal-statistical average:

4 $$\left(\hat{O}\right)\equiv \frac{\sum _{l}\,{e}^{-\beta {\varepsilon }_{l}}\langle l|\hat{O}|l\rangle }{{\mathscr{Z}}},$$

where *ε*_*l*_ is the *l*-th eigenvalue of *H*^*MF*^, |*l*〉 is the corresponding eigenvector, and the partition function of the system is given by ${\mathscr{Z}}={\sum }_{l}\,\exp (\,-\,\beta {\varepsilon }_{l})$ with *β* = 1/*k*_*B*_*T* being the inverse temperature.

The numerical procedures for self-consistent calculations proceed as follows: Suppose we are given a fixed chemical potential *μ*; Firstly, we initialize the Hamiltonian in Eq. (3) with initial distributions of the charge order parameter Θ and superfluid orders *ψ*_*e*_ and *ψ*_*o*_; Secondly, we diagonalize the Hamiltonian to obtain eigenvalues and associated eigenfunctions which are expressed in terms of linear combinations of basis vectors $|{n}_{e},{n}_{o}\rangle$; Finally, we compute the expectations values of operators *b*_*e*_, *b*_*o*_, *n*_*e*_ and *n*_*o*_ to obtain the values of the order parameters, so that a self-consistent calculation can be performed. For calculations with a fixed filling factor *f*, it is a little cumbersome as one needs to relax *μ* and to fixed $\langle {n}_{e}+{n}_{o}\rangle$.

The internal energy of the supercell can be evaluated as $E=\left({\hat{H}}^{MF},+,\mu ,\left({\hat{n}}_{e},+,{\hat{n}}_{o}\right)\right)$. The specific heat can be extracted from the numerical derivative of the internal energy with respect to the temperature ${C}_{V}={(\partial E/\partial T)}_{\mu ,V}$. For the entropy, it can be readily evaluated from the standard statistical relation $S={k}_{B}{\sum }_{l}\,{p}_{l}\,\mathrm{ln}\,{p}_{l}$ with ${p}_{l}={e}^{-\beta {\varepsilon }_{l}}/{\mathscr{Z}}$.

## Calculation and Results

Before embarking on a detailed study on finite-temperature properties of the system, we consider the system at zero temperature and at the atomic limit where *zJ*/*U* = 0. Since the superfluid order parameters vanish in this case, the mean-field Hamiltonian reduces to

5 $${\hat{H}}^{MF}=\sum _{s=e,o}\,\frac{U}{2}[{\hat{n}}_{s}({\hat{n}}_{s}-1)-\mu {\hat{n}}_{s}]-2K\Theta \left({\hat{n}}_{e},-,{\hat{n}}_{o}\right)+2K{\Theta }^{2}.$$

At zero temperature, with $\Theta =({n}_{e}-{n}_{o})/2$ in mind, the eigenvalue corresponding to eigenvector $|{n}_{e}\rangle |{n}_{o}\rangle$ can be cast as

6 $$\varepsilon \left({n}_{e},,,{n}_{o}\right)=\frac{U}{4}{\left[\left({n}_{e},+,{n}_{o}\right)-\left(1+\frac{2\mu }{U}\right)\right]}^{2}+\frac{U}{4}\left[\left(1-\frac{2K}{U}\right){({n}_{e}-{n}_{o})}^{2}-{\left(1+\frac{2\mu }{U}\right)}^{2}\right].$$

**Figure 1:**
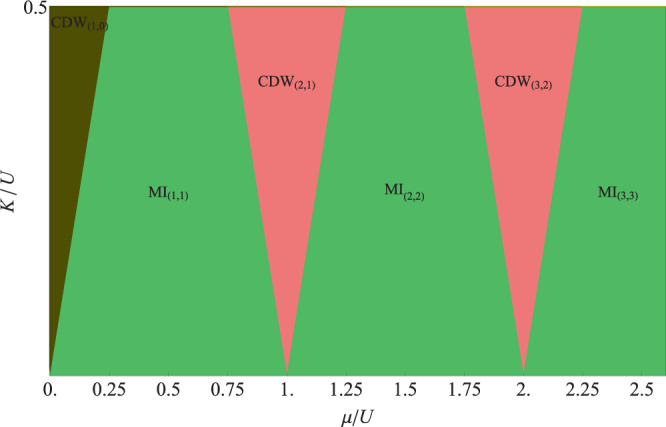
Ground-state phase diagram spanned by *μ*/*U* and *K*/*U* in the atomic limit where *zJ*/*U* = 0 and *K*/*U* ∈ [0, 1/2]. It accommodates two types of incompressible phases: Mott insulating (MI) phases and charge density wave (CDW) phases. The CDW phases are partially polarized in the sense that $|{n}_{e}-{n}_{o}|=1$.

Minimizing the eigenvalue *ε*(*n*_*e*_, *n*_*o*_), one obtains the ground-state phase diagram at the atomic limit. We show the phase diagram in Fig. 1 for the parameter regime *K*/*U* ∈ [0, 1/2]. It features two types of incompressible phases: Mott insulating phases (MI) and charge density wave phases (CDW). The Mott phase MI_(*ne*, *no*)_ is characterized by equal population on even site and odd site with *n*_*e*_ = *n*_*o*_, while the CDW_(*ne*, *no*)_ phase is characterized by unequal population on even site and odd site with *n*_*e*_ ≠ *n*_*o*_. We may always assume that *n*_*e*_ ≥ *n*_*o*_ as the system enjoys a ${{\mathbb{Z}}}_{2}$ symmetry.

**Figure 2:**
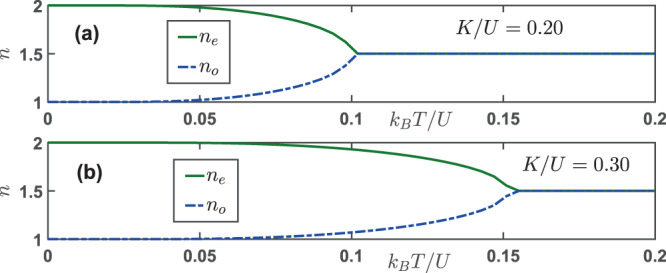
The number densities at even lattice site *n*_*e*_ and odd lattice site *n*_*o*_ as a function of varying temperatures for (**a**) *K*/*U* = 0.20 and (**b**) *K*/*U* = 0.30. At zero temperature, the system is in the CDW_(2,1)_ phase with a charge order. There exists a critical temperature above which the charge order is melted out. Increasing the global-range interaction strength *K*/*U* tends to sustains the charge order against thermal fluctuations. The parameters used here are: *zJ*/*U* = 0 and *μ*/*U* = 1.0.

The effects of finite temperatures on a charge density wave is shown in Fig. 2. In panel (a) where *K*/*U* = 0.2, at zero temperature the system is in the *CDW*_(2,1)_ phase. As the temperature goes up, the density on even site *n*_*e*_ decreases from an integer value, while the density on odd site *n*_*o*_ increases. There exists a critical temperature at which the density on both sites become equal with *n*_*e*_ = *n*_*o*_, indicating that the charge order parameter Θ vanishes. During the process, we have kept the chemical potential to be fixed at *μ*/*U* = 1.0, and the total density *n* = *n*_*e*_ + *n*_*o*_ is almost a constant. In panel (b) where *K*/*U* = 0.3, the trend is similar as in panel (a), except that the critical temperature above which the charge order becomes zero increases from 10 *U*/*k*_*B*_ to approximately 0.15 *U*/*k*_*B*_. This fact suggests that for a CDW phase increasing the global-range interaction strength enhances the charge order by increasing the critical temperature.

**Figure 3:**
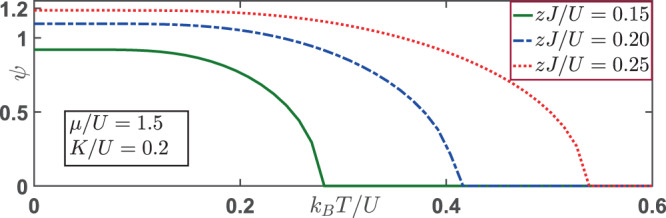
The superfluid order parameter *ψ* = *ψ*_*e*_ = *ψ*_*o*_ as a function of varying temperatures for different values of hopping parameter *zJ*/*U*. At zero temperature, the system is in the SF state with nonzero *ψ* and vanishing charge order. Across the transition temperature, the system undergoes a continuous phase transition from a superfluid state to a normal state. A larger hopping amplitude corresponds to a larger superfluid transition temperature. The parameters used here are: *K*/*U* = 0.2 and *μ*/*U* = 1.5.

Now we take the effects of a finite hopping amplitude into account as well. At sufficient magnitude of hopping parameter *zJ*/*U*, one expects that the system possesses superfluidity with a nonzero order parameter *ψ*. We show the behaviors of the order parameter *ψ* as a function of varying temperatures *k*_*B*_*T*/*U* for different hopping parameters *zJ*/*U* in Fig. 3. At zero temperature and *μ*/*U* = 1.5, the system is in the phase of *MI*_(2,2)_ with a vanishing charge order, as can be read from Fig. 1. Now a sufficiently large hopping amplitude (*zJ*/*U* = 0.15) gives rise to a homogeneous superfluid state with *ψ*_*e*_ = *ψ*_*o*_. As the temperature increases, the superfluid order parameter decreases gradually, and eventually the superfluid order parameter *ψ*_*e*_ vanishes above the transition temperature *T*_*c*_. It is evident that a larger hopping amplitude leads to a larger transition temperature. When the magnitude of the global-range interaction is changed to *K*/*U* = 0.3, our numerical results doesn’t get modified. This is expected as the effective Hamiltonian in Eq. (3) for Θ = 0 reduces to two decoupled conventional Bose-Hubbard models at even and odd sites.

**Figure 4:**
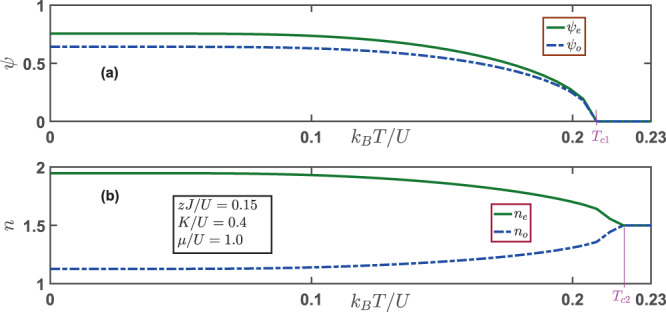
Temperature dependence of (**a**) the superfluid order parameters *ψ*_*e*_ and *ψ*_*o*_ and (**b**) the number densities *n*_*e*_ and *n*_*o*_. At zero temperature, the system is in the supersolid phase with both superfluid order and charge density order. By increasing the temperature, the superfluid order disappears at a critical temperature *k*_*B*_*T*_*c*1_/*U* = 0.209, while the charge order melts out at a higher temperature *k*_*B*_*T*_*c*2_/*U* = 0.219. The parameters used here are: *μ*/*U* = 1.0, *K*/*U* = 0.4 and *zJ*/*U* = 0.15.

We proceed to consider the effects of finite temperatures on the supersolid phase, where both superfluid order and charge order are present. As shown in Fig. 4(a), with the increasing of the temperature, both the superfluid order parameter *ψ*_*e*_ and *ψ*_*o*_ decrease. When the temperature reaches a certain value, the system becomes a conventional superfluid with *ψ*_*e*_ = *ψ*_*o*_. As the temperature is increased further to *T*_*c*1_, the system enters into a CDW state with vanishing superfluid order parameter. Meanwhile, the number densities at both sites display striking behaviors in Fig. 4(b). The number density *n*_*e*_ decreases as the temperature goes up, while the number density *n*_*o*_ increases correspondingly, demonstrating that the transferring of the particles from sites of high population to sites of low population due to the increasing of temperature. At the temperature rises to the superfluid transition temperature *T*_*c*1_, there still exists some residual charge order, which is destroyed completely only after the temperature is lifted to a higher critical temperature *T*_*c*2_.

**Figure 5:**
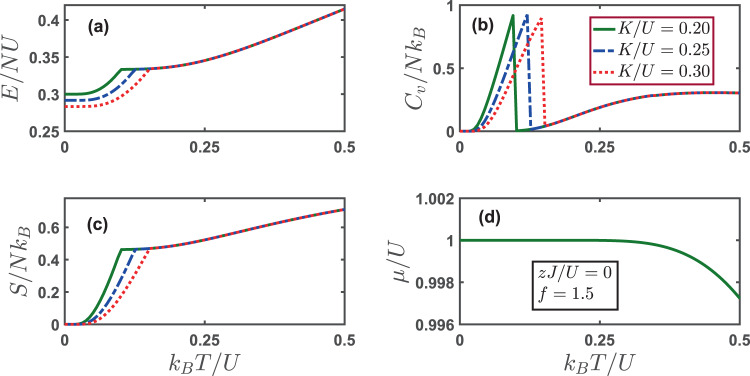
(**a**) Energy per particle *E*/*NU* (**b**) specific heat per particle *C*_*V*_/*Nk*_*B*_ (**c**) entropy per particle *S*/*Nk*_*B*_ and (**d**) chemical potential as a function of varying temperatures for different global-range interaction strength *K*/*U*. At zero temperature, the system is in the phase of *CDW*_(2,1)_. The parameters used here are: *zJ*/*U* = 0 and the filling factor *f* = 1.5.

We turn our attention to the thermodynamics of the system at a fixed filling factor *f* = (*n*_*e*_ + *n*_*o*_)/2. We shall follow the sequences as we study the temperature dependence of the superfluid order parameters and number densities. For the charge density discussed in Fig. 2, its thermodynamic functions such as energy, specific heat, entropy and chemical potential are shown in Fig. 5. The energy per particle *E*/*NU* increases steadily with the temperature *k*_*B*_*T*/*U*. It is remarkable that a larger global-range interaction *K*/*U* leads to a lower energy below the transition temperature. At the transition point where the charge order is completely melted, the specific heat *C*_*V*_/*Nk*_*B*_ shows a characteristic cusp. The entropy per particle *S*/*Nk*_*B*_ starting from zero increases monotonically with the temperature, a sign of increasing disorder. Interestingly, the chemical potential decreases slightly with increasing temperatures and does not depend on the strength of global-range interaction. When the temperature is sufficiently high, the thermodynamics of the system is immune to the strength of global-range interaction *K*/*U*.

**Figure 6:**
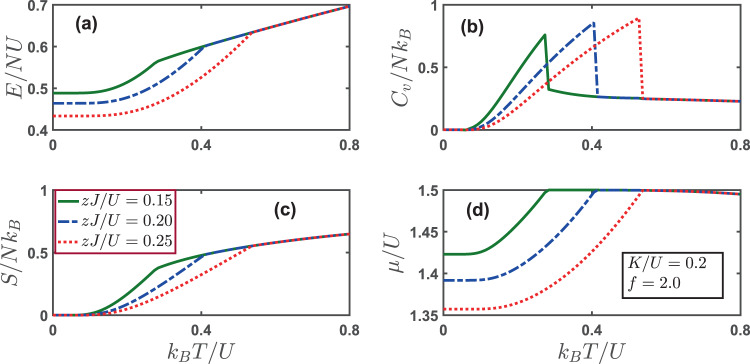
(**a**) Energy per particle *E*/*NU* (**b**) specific heat per particle *C*_*V*_/*Nk*_*B*_ (**c**) entropy per particle *S*/*Nk*_*B*_ and (**d**) chemical potential as a function of varying temperatures for different hopping parameters *zJ*/*U*. At zero temperature, the system is in the superfluid state with Θ = 0. The parameters used here are: *K*/*U* = 0.2 and the filling factor *f* = 2.

We continue to consider the thermodynamics of conventional superfluid state. The temperature dependence of relevant order parameters is revealed in Fig. 3. Here we show the behaviors of energy, specific heat, entropy and chemical potential in Fig. 6. As can be seen in panel (a), the energy per particle *E*/*NU* increases monotonically with the temperatures. It is intuitive to notice that a larger hopping amplitude *zJ*/*U* leads to a lower energy. However, it is consistent with the behavior of entropy per particle *S*/*Nk*_*B*_ shown in panel (c). The entropy increases as the temperature gets higher, with a larger hopping amplitude *zJ*/*U* corresponding to a smaller entropy. This is due to the fact that a larger hopping amplitude enhances superfluidity, leading to an ordered phase with a lower entropy. The specific heat per particle *C*_*V*_/*Nk*_*B*_ shows a nonmonotonic behavior. It exhibits a peak at the transition temperature, indicating the disappearance of the superfluid order. At low temperatures, the chemical potential increases sharply until it reaches a maximum at the transition, with a larger hopping amplitude corresponding to a lower chemical potential. At sufficient high temperature, the thermodynamics of the system is immune to the strength of hopping amplitude *zJ*/*U*.

**Figure 7:**
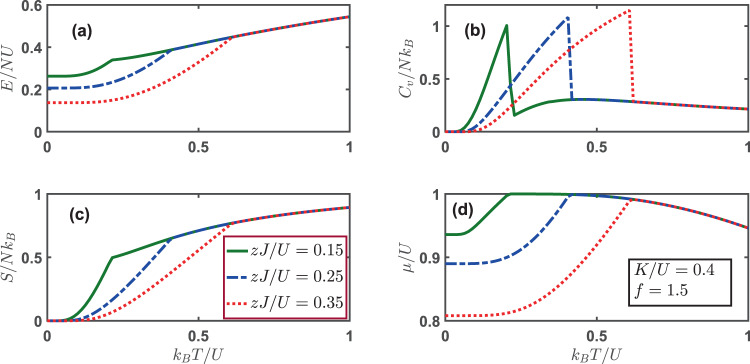
(**a**) Energy per particle *E*/*NU* (**b**) specific heat per particle *C*_*V*_/*Nk*_*B*_ (**c**) entropy per particle *S*/*Nk*_*B*_ and (**d**) chemical potential as a function of varying temperatures for different hopping parameters *zJ*/*U*. At zero temperature, for *zJ*/*U* = 0.15, the system is in the supersolid phase, while for *zJ*/*U* = 0.25 and *zJ*/*U* = 0.35 the system is in the SF state. The parameters used here are: *K*/*U* = 0.4 and the filling factor *f* = 1.5.

Finally, we turn our focus to the thermodynamics for a supersolid phase. As shown in Fig. 7, the supersolid phase only exists in a limited regime of phase space, which is *zJ*/*U* ∈ (0.13, 0.20) in our case. Our numerical solution indicates that for *zJ*/*U* = 0.25 and *zJ*/*U* = 0.35, the system is in the conventional superfluid phase absent of the charge order. The energy per particle *E*/*NU* follows a monotonically increasing trend for all three typical values of *zJ*/*U*. The specific heat per particle *C*_*v*_/*Nk*_*B*_ exhibits a characteristic cusp at the transition temperature at which the superfluid order parameter vanishes. The entropy per particle *S*/*Nk*_*B*_ increases with temperature, indicating tendency toward disorder. The chemical potential manifests a nonmonotonic behavior with the maximum occurring at the transition temperature, and drops gradually with increasing temperature.

## Summary

To sum up, we have studied the extended Bose-Hubbard model with global-range interactions at finite temperatures. We formulated a self-consistent mean-field theory to describe the finite-temperature physics. We have obtained temperature dependence of superfluid order parameters and number densities on even and odd lattice sites. Remarkably, we find that the melting of the charge order is gradually happened as the temperature is increased. For thermodynamic behaviors, we show the variations of energy, specific heat and entropy per particle with the varying of the temperatures and some external tuning parameters. Interestingly, we demonstrate that specific heat show characteristic behaviors across the phase transition. Our results help to establish a qualitative picture for this system at finite temperatures, which are interesting from experimental point view. We expect that such study will stimulate further interesting works from both theoretical side and experimental side.
